# Recurrent Shigella flexneri Gastroenteritis With Concurrent Rotavirus and SARS-CoV-2 Detection in a Preterm Infant: A Case Report

**DOI:** 10.7759/cureus.109013

**Published:** 2026-05-17

**Authors:** Filippos Filippatos, Konstantinos Kakleas

**Affiliations:** 1 Infectious Diseases and Chemotherapy Research Laboratory, First Department of Pediatrics, Medical School, National and Kapodistrian University of Athens, Aghia Sophia Children’s Hospital, Athens, GRC; 2 Department of Allergy and Immunology, Aghia Sophia Children’s Hospital, Athens, GRC

**Keywords:** household transmission, infant, prematurity, recurrence, rotavirus detection, sars-cov-2, shigella flexneri

## Abstract

We report a six-month-old male infant born late preterm at 35 weeks’ gestation who presented with fever, moderate dehydration, and bloody mucoid diarrhea. Stool culture yielded *Shigella flexneri*, while gastrointestinal multiplex testing also detected rotavirus and SARS-CoV-2. Only *S. flexneri* was culture-confirmed and clinically concordant with dysentery; rotavirus vaccine-strain shedding and incidental SARS-CoV-2 detection could not be excluded. The child improved with rehydration, intravenous cefotaxime, followed by oral azithromycin, and supportive care. Two later episodes at 15 and 18 months again yielded *S. flexneri* with the same antibiogram and responded to azithromycin. Because rotavirus strain typing, exact vaccination interval, and *Shigella* molecular typing were unavailable, neither viral causality nor relapse versus reinfection could be confirmed. The case emphasizes cautious interpretation of multiplex results, culture-guided therapy, household transmission risk, and infection-control measures in recurrent pediatric enteric infection.

## Introduction

*Shigella* infection is most common in children one to five years of age and is less frequent in early infancy, although severe disease can occur in vulnerable infants [1,2]. Severe bloody diarrhea requires careful assessment of hydration, stool culture when available, and antimicrobial therapy when clinically indicated, with treatment tailored to susceptibility results and local resistance patterns.

Gastrointestinal multiplex assays may detect more than one organism, but detection does not by itself establish pathogenicity [2,3]. After oral rotavirus vaccination, stool positivity may reflect vaccine-strain shedding unless strain-specific testing is performed [4-6]. Similarly, SARS-CoV-2 RT-PCR positivity in children may represent concurrent or recent infection rather than the cause of diarrhea [7].

Recurrent *Shigella* infection in an otherwise immunocompetent infant is clinically important because it raises questions about relapse, reinfection, household transmission, and infection-control gaps. To our knowledge, reports of culture-confirmed *Shigella flexneri* dysentery with concurrent rotavirus and SARS-CoV-2 detection in early infancy, followed by recurrent *S. flexneri* episodes, are extremely limited. We describe this case to highlight cautious interpretation of multiplex results, culture-guided therapy, and consideration of household transmission when recurrent shigellosis occurs.

## Case presentation

A six-month-old male infant born at 35 weeks’ gestation by spontaneous vaginal delivery (birth weight 2.4 kg; Apgar scores 8 and 9) presented with 24 hours of fever, irritability, poor feeding, lethargy, and 10-12 watery bloody mucoid stools per day. There was no vomiting, cough, or respiratory distress, and urine output had decreased markedly. He had an uncomplicated neonatal course and normal growth at presentation (weight 6.4 kg, 20th percentile).

He was receiving mixed feeding (breast milk with formula supplementation) and had recently started pureed foods. Immunizations were current, except that only one rotavirus vaccine dose had been administered; the exact interval between vaccination and the index illness could not be verified retrospectively. The available record documented mixed feeding and pureed foods but did not reliably document all hygiene/sanitation details, including who prepared feeds, feeding vessel, water source, and diaper-disposal practices. A three-year-old sibling attending daycare had self-limited diarrhea two weeks earlier.

On examination, the infant appeared ill and mildly lethargic but arousable. He had dry mucous membranes, sunken eyes, delayed capillary refill, and an estimated 8% weight loss, consistent with moderate dehydration. Temperature was 38.3°C, heart rate 160/min, respiratory rate 38/min, blood pressure 92/56 mmHg, and oxygen saturation 98% on room air. The abdomen was mildly distended but soft and non-tender, without hepatosplenomegaly. Chest, cardiovascular, and neurologic examinations were otherwise unremarkable.

Initial laboratory studies showed leukocytosis (white blood cell count 18.2 × 10⁹/L, 80% neutrophils), hemoglobin 10.8 g/dL, thrombocytosis (610 × 10⁹/L), elevated C-reactive protein (CRP 60 mg/L), mild hyponatremia (130 mmol/L), hypokalemia (3.3 mmol/L), mild metabolic acidosis, alanine aminotransferase (ALT 95 U/L), and aspartate aminotransferase (AST 80 U/L). Stool microscopy showed abundant leukocytes and erythrocytes; renal function was normal.

Stool culture yielded *S. flexneri* susceptible to cefotaxime, ciprofloxacin, azithromycin, and gentamicin and resistant to ampicillin and trimethoprim-sulfamethoxazole; no extended-spectrum beta-lactamase (ESBL) phenotype was detected. Susceptibility interpretation followed the reporting laboratory's standard criteria. Rotavirus was detected by gastrointestinal multiplex PCR, and SARS-CoV-2 was detected by rapid antigen testing and RT-PCR (cycle threshold (Ct) 28). The Ct value documented SARS-CoV-2 RNA detection but did not establish causality for gastrointestinal symptoms. Because rotavirus strain typing or sequencing was not performed and the vaccination interval was unavailable, vaccine-strain shedding could not be excluded. In the absence of respiratory symptoms, SARS-CoV-2 detection was regarded with caution as a concurrent finding rather than an established cause of the gastrointestinal manifestation. Blood and urine cultures were negative.

The infant was admitted under contact isolation. He received a 20 mL/kg bolus of 0.9% saline followed by intravenous rehydration with potassium supplementation, then oral rehydration when tolerated. Intravenous cefotaxime (150 mg/kg/day in three doses) was started empirically and continued for 72 hours. After clinical improvement and confirmation of susceptibility, treatment was switched to oral azithromycin (10 mg/kg/day) for two additional days to complete a five-day antibiotic course. Zinc sulfate (10 mg/day for 10 days) and supportive care were also given.

Stools remained loose during the first 24 hours, but visible blood had resolved by day 3. By day 5, sodium and potassium had normalized, CRP had fallen to 20 mg/L, and the leukocyte count to 11.5 × 10⁹/L. The infant was discharged from the hospital on day 6 in good health with instructions on hand hygiene, safe diaper disposal, separate feeding utensils, and signs of dehydration. Stool testing of household contacts was suggested but not carried out, limiting assessment of a possible household reservoir.

At 15 months, he returned with low-grade fever, irritability, and recurrent bloody diarrhea (6-8 stools/day). Laboratory tests indicated a white blood cell count of 15.4 × 10⁹/L, CRP 40 mg/L, and mild hyponatremia. Stool culture again yielded *S. flexneri* with the same antibiogram as the initial isolate; multiplex viral PCR was negative for rotavirus, adenovirus, norovirus, and SARS-CoV-2. Symptoms resolved after oral azithromycin (10 mg/kg/day for 5 days) and oral rehydration.

**Figure 1:**
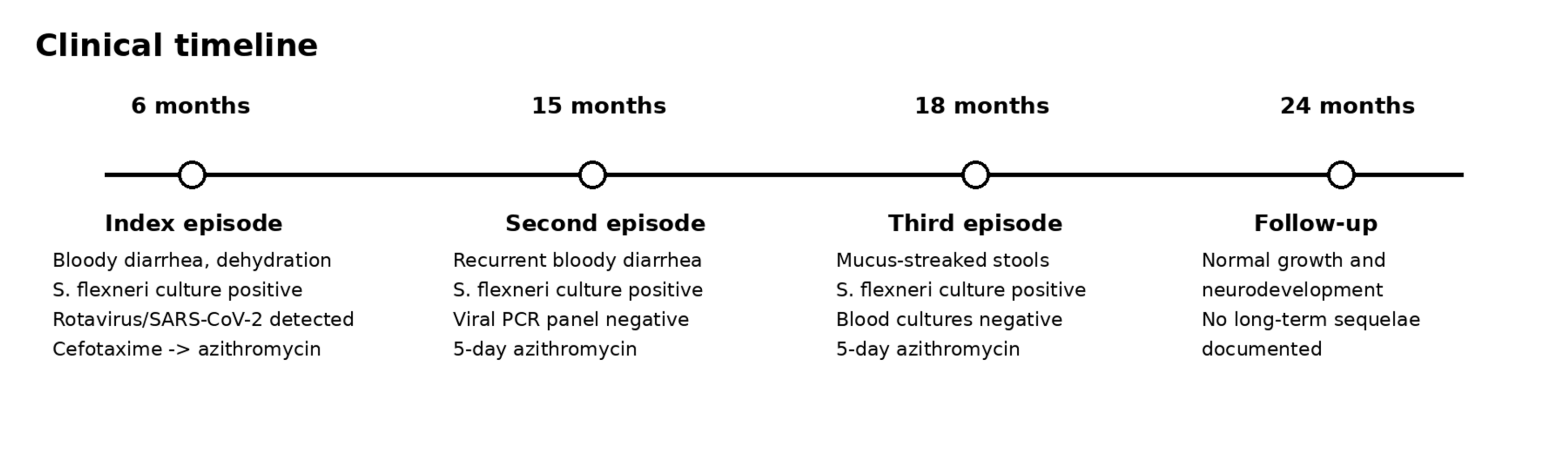
Clinical timeline of index and recurrent episodes, microbiological findings, treatment, and follow-up. Abbreviation: *S. flexneri*, *Shigella flexneri.*

At 18 months, a third episode occurred with moderate diarrhea and mucus-streaked stools but no dehydration. Stool culture again isolated *S. flexneri* with the same resistance profile. Blood cultures were negative, and CRP was 30 mg/L. The child again improved with a five-day azithromycin course and supportive care. Growth and neurodevelopment remained normal throughout follow-up to 24 months. Laboratory findings and *S. flexneri* antibiograms across the three episodes at 6, 15, and 18 months are presented in Tables 1-2. Clinical timeline of index and recurrent episodes, microbiological findings, treatment, and follow-up is presented in Figure 1.

**Table 1:** Key laboratory findings across the three episodes at 6, 15 and 18 months, respectively. Note: Values were recorded at presentation for each episode unless otherwise stated. Reference ranges are age-adjusted and may vary by laboratory. Bold font indicates values outside the stated reference range. Abbreviations: ALT, alanine aminotransferase; AST, aspartate aminotransferase; CRP, C-reactive protein; WBC, white blood cell count.

Parameter	Episode 1	Episode 2	Episode 3	Reference range
	(6 months; index episode)	(15 months)	(18 months)	
WBC (×10⁹/L)	18.2	15.4	13.7	6-15 × 10⁹/L
Hemoglobin (g/dL)	10.8	11.5	11.8	11.0-13.5 g/dL
Platelets (×10⁹/L)	610	520	530	150-450 × 10⁹/L
CRP (mg/L)	60	40	30	<5 mg/L
ALT (U/L)	95	45	40	≤40 U/L
AST (U/L)	80	42	38	≤40 U/L
Sodium (mmol/L)	130	135	134	135-145 mmol/L
Potassium (mmol/L)	3.3	3.6	3.7	3.5-5.0 mmol/L

**Table 2:** Shigella flexneri antimicrobial susceptibility results across the three episodes at 6, 15, and 18 months of age. Note: The *Shigella flexneri* isolates from all three episodes showed the same susceptibility profile: susceptible to cefotaxime, ciprofloxacin, azithromycin, and gentamicin; resistant to ampicillin and trimethoprim-sulfamethoxazole; and without an ESBL phenotype. MIC values are in mg/L. Interpretive criteria are shown as susceptible/intermediate/resistant where applicable and should be interpreted according to the reporting laboratory method and local standard. *Azithromycin interpretive criteria for enteric pathogens may vary by standard and region; therefore, the azithromycin MIC should be interpreted cautiously and in clinical context. Abbreviations: ESBL, extended-spectrum beta-lactamase; I, intermediate; MIC, minimum inhibitory concentration; R, resistant; S, susceptible; TMP-SMX, trimethoprim-sulfamethoxazole.

Antimicrobial agent/phenotype	Episode 1 (6 months)	Episode 2 (15 months)	Episode 3 (18 months)	Interpretive criteria shown (S/I/R; mg/L)
Ampicillin	R (MIC >16)	R (MIC >16)	R (MIC >16)	≤8/16/≥32
Cefotaxime	S (MIC <1)	S (MIC <1)	S (MIC <1)	≤1/2/≥4
Ciprofloxacin	S (MIC <0.06)	S (MIC <0.06)	S (MIC <0.06)	≤0.25/0.5/≥1
Azithromycin*	S (MIC 4)	S (MIC 4)	S (MIC 4)	≤8/16/≥32*
TMP-SMX	R (MIC >4/76)	R (MIC >4/76)	R (MIC >4/76)	≤2/38/ - /≥4/76
Gentamicin	S (MIC <4)	S (MIC <4)	S (MIC <4)	≤4/8/≥16
ESBL phenotype	Negative	Negative	Negative	Not applicable

## Discussion

This case is best interpreted as recurrent culture-confirmed *S. flexneri* dysentery in an infant born late preterm, with concurrent detection of rotavirus and SARS-CoV-2 during the index episode. Although three pathogens were detected, only *S. flexneri* was culture-confirmed and consistent with the clinical presentation of dysentery. The contribution of rotavirus and SARS-CoV-2 remains uncertain because rotavirus vaccine-strain shedding and incidental or recent SARS-CoV-2 infection could not be excluded. This cautious interpretation is consistent with evidence that *Shigella* remains a major cause of pediatric diarrheal disease, that *Shigella* species and serotype distributions vary across populations, that *Shigella* pathogenesis depends on epithelial invasion and inflammatory injury, and that longitudinal birth-cohort data often identify multiple enteric pathogens during childhood diarrhea [1-4].

To our knowledge, reports of concurrent detection of *S. flexneri*, rotavirus, and SARS-CoV-2 in early infancy are extremely limited. However, this case should not be taken as proof of a pathogenic infection involving all detected organisms. Rotavirus is a well-recognized cause of acute diarrhea in young children [5], while pediatric COVID-19 has generally been reported to be milder than adult disease and may be detected without proving causality for gastrointestinal symptoms [6]. In parallel, antimicrobial-resistant *Shigella* has become an important global concern [7]. Molecular studies of pediatric diarrhea show frequent co-detection of multiple enteric pathogens and mixed viral-bacterial findings, emphasizing that multiplex positivity must be interpreted alongside culture results, clinical syndrome, vaccination history, and epidemiologic context [8,9].

Viral-bacterial interactions in the gut are biologically plausible but were not demonstrated in this patient. Rotavirus can damage enterocytes and impair absorption [10]; enteric pathogens may disrupt epithelial barrier function [11]; and SARS-CoV-2 can productively infect human gut enterocytes [12]. ACE2 has intestinal functions linked to amino acid transport, microbial ecology, and inflammation [13], and altered gut microbiota composition has been associated with COVID-19 severity and immune responses [14]. These mechanisms could theoretically amplify inflammation or barrier dysfunction, but the available data in this case do not establish that either virus caused or worsened the *Shigella* episode. These pathways should therefore be considered hypotheses for future investigation rather than explanatory conclusions.

The child's history of late-preterm birth should also be interpreted cautiously. He had no neonatal complications and demonstrated normal growth and neurodevelopment. Age-specific infection vulnerability and interindividual variability are important in early life [15], but at six months of age, late prematurity may have represented only a minor background vulnerability, if any. Evidence on rotavirus vaccination in premature infants also supports careful vaccine-related interpretation rather than assuming that prematurity alone explains severe gastrointestinal disease [16]. More clinically relevant considerations in this patient include incomplete rotavirus immunization, mixed feeding, daycare exposure through an older sibling, and incomplete household transmission assessment.

The recurrence of culture-positive *S. flexneri* at 15 and 18 months with a similar antibiogram suggests either reinfection from a persistent household or environmental source or relapse from an unrecognized reservoir. The absence of whole-genome sequencing prevents a definitive distinction between relapse and reinfection. Household contacts of children with shigellosis may contribute to ongoing transmission [17], and broader analyses of diarrheal disease in children younger than five years highlight the importance of interventions such as sanitation, hygiene, nutrition, vaccination, and other preventive measures [18]. In this case, the fact that stool testing of household contacts was suggested but not completed represents a missed opportunity for transmission analysis. This limitation strengthens the practical public health message regarding hand hygiene, diaper disposal, disinfection of shared surfaces, and contact evaluation when recurrent shigellosis occurs.

The inflammatory-marker trajectory was consistent with clinically improving bacterial dysentery: CRP declined from 60 mg/L at the index episode to 20 mg/L by day 5 after treatment, and later episodes were associated with lower CRP values of 40 mg/L and 30 mg/L. The mild ALT/AST elevation during the index episode was nonspecific; there was no hepatosplenomegaly or separate hepatobiliary diagnosis documented. These findings should be interpreted as supportive clinical context rather than evidence of a specific mechanism.

Antimicrobial management was guided by severity, culture results, and susceptibility testing. Cefotaxime was used initially because the infant was ill and moderately dehydrated, and treatment was narrowed to oral azithromycin once the child improved and susceptibility was confirmed. The total antibiotic duration for the index episode was five days, and the two recurrent episodes were treated with five-day azithromycin courses. Future prevention is also important: broad-spectrum *Shigella* vaccine strategies continue to be investigated [19], while pediatric data from multiple settings show increasing antimicrobial resistance and the need for ongoing susceptibility surveillance [20]. Because azithromycin interpretive criteria and resistance patterns can vary by region and laboratory standard, culture confirmation, susceptibility testing, and surveillance remain important.

This case has several limitations. Rotavirus strain typing was not performed, the exact timing of rotavirus vaccination could not be verified, and SARS-CoV-2 sequencing was unavailable. *Shigella* isolates were not whole-genome sequenced, so relapse and reinfection cannot be distinguished definitively. Hygiene/sanitation details and household contact testing were incomplete, and cytokine or microbiome profiling was not performed. The report is, therefore, hypothesis-generating and should not be generalized beyond the clinical observations described.

## Conclusions

Culture-confirmed *S. flexneri* dysentery with concurrent rotavirus and SARS-CoV-2 detection, followed by recurrent *S. flexneri* isolation, is unusual in early infancy. This case underscores that multiplex positivity should be interpreted with culture results, symptoms, vaccination history, and epidemiologic context. It also highlights the importance of culture-guided therapy, household infection-control measures, contact evaluation when feasible, and surveillance for resistant *Shigella*. Mechanistic links between co-detected viruses and recurrent *Shigella* infection remain hypotheses for future study.
